# Cyclodextrin-induced host–guest effects of classically prepared poly(NIPAM) bearing azo-dye end groups

**DOI:** 10.3762/bjoc.8.224

**Published:** 2012-11-14

**Authors:** Gero Maatz, Arkadius Maciollek, Helmut Ritter

**Affiliations:** 1 Institute of Organic Chemistry and Macromolecular Chemistry, Heinrich-Heine-University Duesseldorf, Universitaetsstraße 1, D-40225 Duesseldorf, Germany

**Keywords:** azo-dye, cyclodextrins, end-group functionalization, host–guest interaction, supramolecular aggregation

## Abstract

A thermo-, pH- and cyclodextrin- (CD) responsive poly(*N*-isopropylacrylamide) (PNIPAM), with a *N*,*N*-dimethylaminoazobenzene end group was synthesized. Using 3-mercaptopropionic acid as a chain transfer agent, PNIPAM with a well-defined COOH end group was obtained. The acid end group was transferred to the corresponding acid chloride and then functionalized with *N*,*N*-dimethyl[4-(4’-aminophenylazo)phenyl]amine. This dye-end-group-labeled polymer showed acidochromic effects, depending on the pH and the presence of randomly methylated β-cyclodextrin (RAMEB-CD). Also higher cloud-point values for the lower critical solution temperature (LCST) in the presence of RAMEB-CD were observed. Additionally, this azo-dye-end-group-labeled polymer was complexed with hyperbranched polyglycerol (HPG) decorated with β-CD to generate hedgehog-like superstructures.

## Introduction

Polymers bearing dyes, such as azo, stilbene, anthraquinone or fluorescence dyes, in the main or side chain have been widely described and investigated [1–15]. A few examples of dyes located at the end group of polymers are prepared preferably under anionic or controlled conditions such as reversible addition–fragmentation chain-transfer polymerization (RAFT) or atom-transfer radical polymerization (ATRP) [14–16]. However, up to now, only a little is known about the preparation of dye-end-group-labeled polymers by using classical free-radical polymerization techniques. Water-soluble polymers, which exhibit a lower critical solution temperature (LCST), e.g., many poly(*N*-alkylacrylamides), have found numerous practical applications in waterborne smart materials such as bioactive surfaces, selective bioseparation, or hyperthermia-induced drug delivery [14]. Several reports are available on the preparation and properties of thermally responsive polyacrylamides containing azobenzene or stilbene dyes in the side chain [3,14]. Furthermore, the interaction of dye-containing polymers with CD in water is of some interest, because of their external, light-induced, reversible complexation [1–3,10–11]. In recent years, increasing attention has been given to supramolecular structures, the science of noncovalent assembly in biological systems and chemical processes [12]. Hyperbranched polymers such as polyglycerols (HPG), as a result of their inherent dendritic topology, attract considerable interest for a wide range of optical, medical or reagent-immobilization applications [16].

## Results and Discussion

**Scheme 1:**
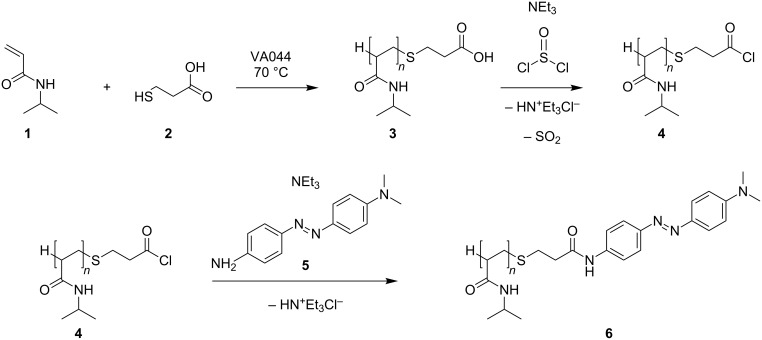
CTP of **1** and end-group functionalization with **5** yielding the azo-dye-end-group-labeled polymer **6**.

Here, we describe the free-radical polymerization of *N*-isopropylacrylamide (NIPAM, **1**) in the presence of a chain–transfer agent and its end-group functionalization by condensation with an azo dye. We also investigated the host–guest interaction of the azo-dye-labeled end group with randomly methylated β-cyclodextrin (RAMEB-CD), and with HPG bearing β-CD on top. Thus, the focus of the present study was directed towards the preparation and superstructure formation of a thermo- and pH-responsive polymer bearing an azo dye at the end group. In a first step, a chain-transfer polymerization (CTP) of *N*-isopropylacrylamide (**1**) was carried out in the presence of 3-mercaptopropionic acid (**2**) and with 4,4’-azobis(4-cyanovaleric acid) as initiator, to achieve a high degree of functionalization and yield of carboxy-terminated PNIPAM **3**. Then, the free carboxy end group was transferred in to the corresponding acid chloride by treatment with thionyl chloride, yielding polymers with a propionyl chloride end group (Scheme 1).

The final product **6** was obtained by condensation of **4** with the amino group of the dye *N*,*N*-dimethyl-[4-(4’-aminophenylazo)phenyl]amine (**5**, Supporting Information File 1). The molecular weight (*M*_n_ 3.4 kDa) and molecular weight distribution (D 1.3) of **6** were determined by size-exclusion chromatography (SEC). A *M*_n_ value of **5** was also calculated by end-group analysis based on ^1^H NMR measurements, showing similar results (Supporting Information File 1).

**Figure 1:**
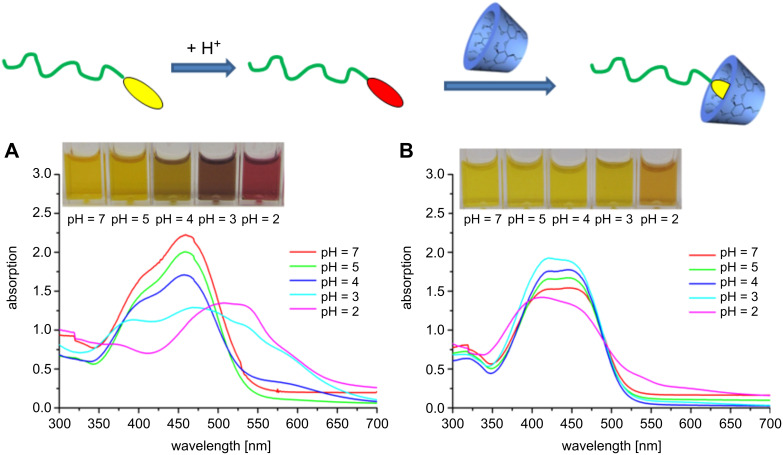
Absorption spectra of **6** in water in a pH range from 7 to 2 (A). Absorption spectra of **6** in water depending on the pH in the presence of RAMEB-CD (B).

The UV–vis absorption spectrum of **6** in water showed the characteristic broad band at λ_max_ 460 nm, which corresponds to the orange color at pH 7 [17]. However, upon decreasing of the pH from 7 to 2, a large bathochromic (red) shift takes place, due to the protonation of the azo dye of **6** [17]. As illustrated in Figure 1A this red shift corresponds to an increase of the absorption up to 500–550 nm, whereas the band at 420 nm decreases. Thus, the protonated azonium tautomer is considered to be the predominant form in the red acidic aqueous solution [17–19].

Due to our general interest in the use of CD in polymer chemistry [3], the interaction of polymer **6** with RAMEB-CD was investigated. The proposed formation of the host–guest structure between the azo-dye end group and the RAMEB-CD cavity was proved by use of 2D ROESY NMR spectroscopy (Supporting Information File 1). The correlation signals between the protons of the RAMEB-CD cavity and the aromatic protons of the azo dye were observed. However, the correlation signals between the RAMEB-CD cavity and the methyl protons of the *N*,*N*-dimethylamine group of the azo dye were not observed. This indicates a complete inclusion of the azo dye inside the CD cavity with an orientation to the RAMEB-CD axis [1]. In addition, UV–vis absorption spectra of **6** were recorded in aqueous solution at a pH range from 7 to 2 and in the presence of RAMEB-CD. Hereby, a strong blue shift in the absorption spectra was observed (Figure 1B). As mentioned above, the absorption spectra of **6** after protonation indicate the formation of the azonium structure (Figure 1A). The subsequent complexation of this protonated azo dye with RAMEB-CD causes a hypsochromic shift. This strongly indicates that, due to the presence of RAMEB-CD, a shift of the equilibrium from the azonium to the amonium form of the azo dye takes place. Consequently, the azo group in **6** is supramolecularly protected from protonation by the surrounding CD ring, while the free dimethylamino group is easily protonated. These results correspond to the work of Toda et al. [19].

**Figure 2:**
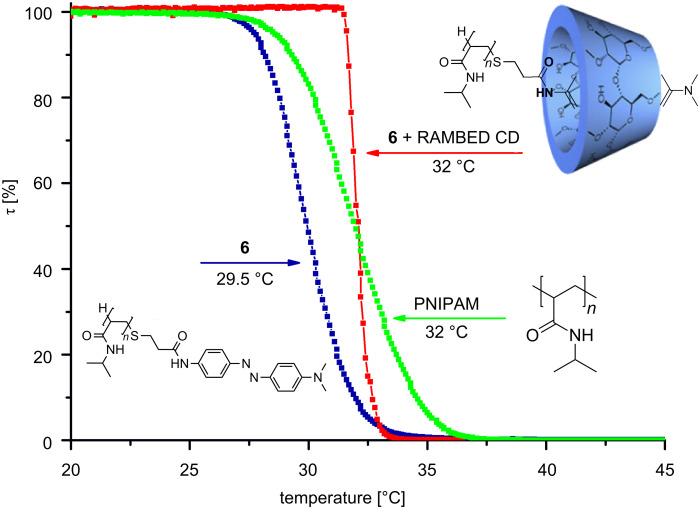
LCST measurements of **6**, the complex of **6** and RAMEB-CD, and in comparison to pure PNIPAM.

Furthermore, the solution properties of polymer **6**, depending on the temperature in the presence of RAMEB-CD, were investigated. As expected, **6** is soluble in cold water below the critical solution temperature (LCST). However, due to the hydrophobic azo-dye end group of **6**, a slight reducing effect on the cloud point in comparison to pure PNIPAM, from 32 down to 29.5 °C is detected. As a result of complexation of the hydrophobic azo-dye end group by RAMEB-CD, the cloud point increases from 29.5 back to 32 °C (Figure 2).

**Figure 3:**
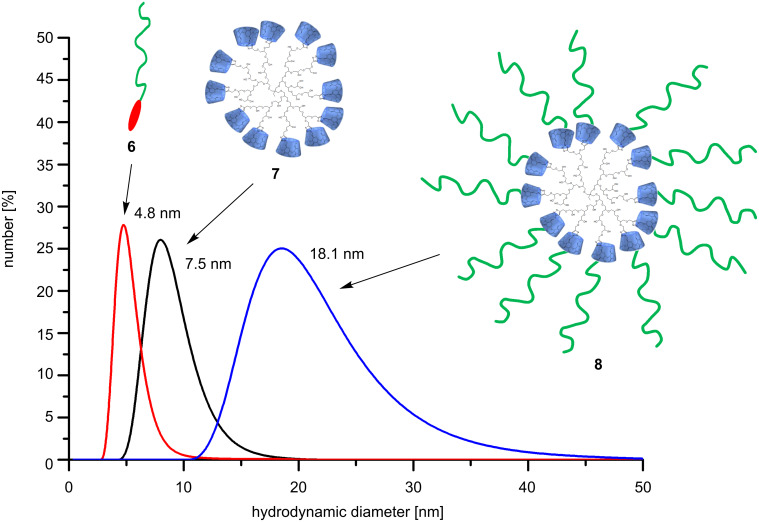
Hydrodynamic diameters of **6**, **7** and **8** (1 mg/mL) at 20 °C.

This is strong evidence for the interaction between the hydrophobic azo-dye end group and the hydrophilic RAMEB-CD ring. Based on this experience, the complexation of the azo-dye end group of **6** with hyperbranched polyglycerol (HPG, **7**) decorated with β-CD was investigated. This kind of supramolecular structure was generated to illustrate the broad applicability of **6** to generate, e.g., hedgehog-like nanoshapes. Therefore, **7** was prepared according to a procedure described in the literature recently [20–22]. The molecular weight of **7** (*M*_n_ 38 kDa) and the dispersity (D 1.7) were determined by SEC (Supporting Information File 1). To confirm the complexation between the azo-dye-labeled polymers **6** and **7** and the formation of a hedgehog-like superstructure **8**, DLS measurements in water were carried out. As expected, the complexation of **6** by **7** leads to an increase of the hydrodynamic diameters up to 18 nm (Figure 3).

**Figure 4:**
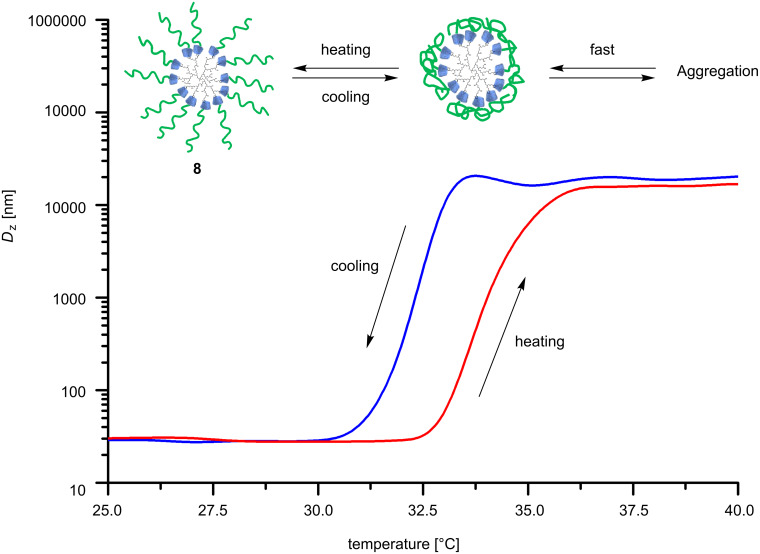
z-Average diameter (*D*_Z_) of the complex **8** in water as a function of temperature (0.5 mg/mL, heating rate 0.5 °C/min).

The aggregation behavior of complex **8** in aqueous solution was also investigated by DLS measurements. Figure 4 shows the temperature dependence of the z-average diameter (*D*_Z_) of the hedgehog-like nanoshaped polymer **8**. In the temperature range from 25 to 32 °C no change of *D*_Z_ was recorded.

At 33 °C the *D*_Z_ of **8** increased up to 21000 nm, but the values showed poor stability because of sedimentation. This means that **8** is able to form larger particles above the LCST, even resulting in precipitation [23]. However, the change of *D*_Z_ showed a good reversibility upon cooling. The behavior can be interpreted by the hydrophobic shell of **8** and the strong hydrophobic interaction above the LCST, which causes the formation of the large particles.

## Conclusion

In summary, we have presented the synthesis of an azo-dye-end-group-labeled PNIPAM by free-radical polymerization. The acidochromic and thermo-responsive behavior in aqueous solution was analyzed by absorption and cloud-point measurements. In addition, the influences of RAMEB-CD on both effects were investigated. The intermolecular interaction between the dye end group and RAMEB-CD was proven by absorption and 2D ROESY NMR experiments. Furthermore, we were able to show the formation of a hedgehog-like superstructure, based on a hyperbranched polyglycerol, bearing CD moieties, and the end-group-modified PNIPAM **6**.

## Experimental

### General remarks

All reagents used were commercially available (Sigma-Aldrich, Acros Organics) and used without further purification. RAMEB-CD and β-CD were obtained from Wacker Chemie GmbH, Burghausen, Germany and were used after being dried overnight with a vacuum oil pump over P_4_O_10_. *N*,*N*-Dimethylformamide (DMF) was purchased from Fluka, Germany. Dimethylsulfoxide-*d*_6_ (99.9 atom % D) and deuterium oxide, D_2_O were obtained from Deutero GmbH, Germany.

^1^H NMR spectra were recorded on a Bruker Avance DRX 300 at 20 °C by using DMSO-*d*_6_ or deuteriumoxide (99.9%) as solvents. FTIR spectra were recorded on a Nicolet 6700 FTIR spectrometer equipped with an ATR unit. The absorption spectra were measured in 1 cm quartz cells on a Specord 210 Plus UV–visible spectrophotometer (Analytik Jena AG, Germany). SEC-MALS measurements were carried out on a combined system comprising the following elements: refractive-index detector Optilabrex (Wyatt Technologies, laser wavelength 658 nm), three-angle light-scattering detector miniDawn TREOS (Wyatt Technologies, laser wavelength 658 nm, detector angles at 43.5°, 90.0° and 136.5°), UV detector Waters 486 (Waters), column set of HEMAbio 300 and HEMAbio 100 (MZ-Analysentechnik), pump, degasser and autosampler (Agilent 1200, Agilent technologies). The eluent was ultrapure water at a flow rate 1 mL/min. The molecular weight was calculated with Astra5 software from static-light-scattering data, by using the Zimm model. As concentration source, the refractive index was used. Calibration of the system was performed with bovin serum albumin. Turbidity experiments were performed on a Tepper cloud-point photometer TP1. The relative transmission of a laser beam with a wavelength of 670 nm was recorded for each experiment. The measurements were performed in a temperature range between 5 and 60 °C and at a heating rate of 1 °C min^−1^ by using Hellma Suprasil precision cells 110 Q-S. Critical solution temperatures derived from these experiments were determined at 50% relative transmission. Dynamic light scattering (DLS) experiments were carried out with a Malvern Zetasizer Nano; ZS ZEN 3600 at a temperature of 20 °C. The particle size distribution is derived from a deconvolution of the measured intensity autocorrelation function of the sample by a general purpose method, i.e., the non-negative least squares algorithm, included in the DTS software.

**Synthesis of 3. ***N*-Isopropylacrylamide (**1**, 2 g, 0.02 mol) was dissolved in 10 mL of ethanol and flushed with argon for 15 min. To this solution, 3-mercaptopropionic acid (**2**, 0.1 mL, 1 mmol) and 4,4’-azobis(4-cyanovaleric acid) (24.7 mg, 0.09 mmol) were added under an argon atmosphere. After being stirred at 70 °C overnight, the corresponding polymer was separated by precipitation with cold diisopropyl ether. The precipitate was washed three times with diethyl ether and dried under reduced pressure. FTIR (diamond) ν (cm^−1^): 3287 (NH), 2970 (CH), 2930 (CH),1712 (CO), 1640 (CO), 1539 (NH), 1456 (CH), 1365 (CH), 1230 (CH_2_), 1171 (CC); ^1^H NMR (300 MHz, D_2_O) δ 7.19 (br, 1H, NH), 3.85 (br, 1H, CH), 2,67 (br, 2H, CH_2_), 2,4 (br, 2H, CH_2_), 1.3–2.2 (backbone), 1.02 ppm (s, 6H, CH_3_).

**Synthesis of 6.** The carboxy-terminated PNIPAM (**3**, 2 g) was dissolved in 5 mL of dry DMF. To this solution, thionyl chloride (0.15 mL, 2 mmol) and triethylamine (0.28 mL, 2 mmol) were added and stirred for 3 h. The precipitated triethylammonium chloride was filtered and the excess of thionyl chloride was removed in vacuum from the DMF-solution. To the obtained solution, triethylamine (0.28 mL, 2mmol) and *N*,*N*-dimethyl-[4-(4’-aminophenylazo)phenyl]amine (**5**, 360 mg, 1.5 mmol) were added and stirred overnight at room temperature. The triethylammonium chloride was filtered off, and the terminated polymer was isolated by precipitation with cold diisopropyl ether. The precipitate was washed three times with diethyl ether and dried under reduced pressure. FTIR (diamond) ν (cm^−1^): 3287 (NH), 2970 (CH), 2930 (CH), 1712 (CO), 1640 (CO), 1539 (NH), 1456 (CH), 1365 (CH), 1230 (CH_2_), 1171 (CC), 881 (CN), 838 (aromatic C-H, 2 neighboring H atoms); ^1^H NMR (300 MHz, DMSO-*d*_6_) δ 7.97 (d, 8.88 Hz, ArH), 7.98 (d, 7.15 Hz, ArH), 7.58 (d, 7.17 Hz, ArH), 7.36 (d, 8.88 Hz, ArH) 7.20 (br, NH), 3.84 (s, CH), 2.62 (br, CH_2_), 2.46 (br, CH_2_), 1.3–2.2 (backbone), 1.04 ppm (s, 6H, CH_3_); SEC measurements: *M*_n_ 3.4 kDa, *P*_D_ 1.3; ^1^H NMR: 3.3 kDa.

## Supporting Information

The Supporting Information contains experimental procedures for the preparation of **5**, HPG, propargyl-modified HPG, CD-monoazide, HPG bearing β-CD (**7**), and spectroscopic data of **6** and the complex of **6** and RAMEB-CD.

File 1 Experimental procedures and spectroscopic data.
