# Supplementary material for: Cyclodextrin-induced host–guest effects of classically prepared poly(NIPAM) bearing azo-dye end groups
Source: Beilstein J Org Chem. 2012 Nov 14;8:1929–35. doi: 10.3762/bjoc.8.224 (PMC3511033; doi:10.3762/bjoc.8.224)
Supplement: File 1 — Experimental procedures and spectroscopic data. [file Beilstein_J_Org_Chem-08-1929-s001.pdf]

Supporting Information

for

Cyclodextrin-induced host–guest effects of classically prepared poly(NIPAM) bearing azo-dye end groups

Gero Maatz, Arkadius Maciollek and Helmut Ritter*

Address: Institute of Organic Chemistry and Macromolecular Chemistry, Heinrich-Heine-University Duesseldorf, Universitaetsstraße 1, D-40225 Duesseldorf, Germany

Email: Helmut Ritter* - h.ritter@uni-duesseldorf.de

* Corresponding author

Experimental procedures and spectroscopic data

General remarks

All reagents used were commercially available (Sigma-Aldrich, Acros Organics) and were used without further purification. RAMEB-CD and β-CD were obtained from Wacker Chemie GmbH, Burghausen, Germany and were used after drying overnight with a vacuum oil pump over P_4O_{10}.

N,*N*-Dimethylformamide (DMF) was purchased from Fluka, Germany. Dimethylsulfoxide-d_6 (99.9 atom % D) and deuterium oxide, D_2O were obtained from Deutero GmbH, Germany. ^{1}H NMR spectra were recorded on a Bruker Avance DRX 300 at 20 °C with DMSO-d_6 or Deuteriumoxide (99.9%) as solvents. FTIR spectra were recorded on a Nicolet 6700 FTIR spectrometer equipped with an ATR unit. The absorption spectra were measured in 1 cm quartz cells on a Specord 210 Plus UV–visible spectrophotometer (Analytik Jena AG, Germany).

SEC-MALS measurements were carried out on a combined system comprising the following elements: refractive index detector Optilabrex (Wyatt Technologies, laser wavelength 658 nm), three-angle light-scattering detector miniDawn TREOS (Wyatt Technologies, laser wavelength 658 nm, detector angles at 43.5°, 90.0° and 136.5°), UV detector Waters 486 (Waters), column set of HEMAbio 300 and HEMAbio 100 (MZ-Analysentechnik), pump, degasser and autosampler (Agilent 1200, Agilent technologies). The eluent was ultrapure water at a flow rate 1 mL/min. The molecular weight was calculated with Astra5 software from static-light-scattering data, by using the Zimm model. As concentration source, the refractive index was used. Calibration of the system was performed with bovin serum albumin.

Turbidity experiments were performed on a Tepper cloud point photometer TP1. The relative transmission of a laser beam with a wavelength of 670 nm was recorded for

each experiment. The measurements were performed at a temperature range between 5 and 60 °C and a heating rate of 1 °C min^{-1} by using Hellma Suprasil precision cells 110 Q-S. Critical solution temperatures derived from these experiments were determined at 50% relative transmission.

Dynamic light scattering (DLS) experiments were carried out with a Malvern Zetasizer Nano; ZS ZEN 3600 at a temperature of 20 °C. The particle size distribution is derived from a deconvolution of the measured intensity autocorrelation function of the sample by a general purpose method (non-negative least squares) algorithm included in the DTS software.

Synthesis of *N*,*N*-dimethyl[4-(4-nitrophenylazo)phenyl]amine

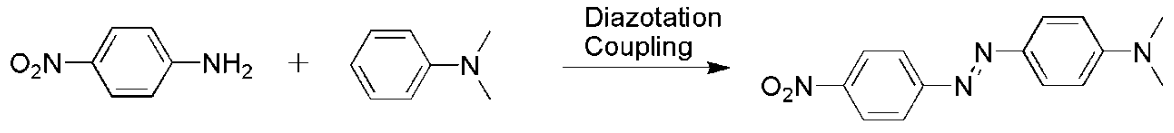

Scheme S1: Synthesis of the red azo-dye **3**.

To a solution of 200 mL water, 30 mL acetonitrile and 20 mL 6 N HCl, 4-nitroaniline (5.16 g, 37.4 mmol) was added portionwise. The solution was cooled down to a temperature below 5 °C. After 10 min of stirring, another solution of sodium nitrite (2.7 g, 39 mmol) and 50 mL water was added dropwise to the first solution. The pH of the reaction was maintained at about 1.5 to 2 by the addition of 6 N HCl. To this cold solution, a solution of *N*,*N*-dimethylaniline (4.8 g, 38 mmol) in 20 mL water was added dropwise. The reaction solution was stirred below 5 °C for 15 min at pH 1.5–2. The solution was basified by adding a solution of K_2CO_3 (4 M). The reaction was allowed to warm to rt over about 2–3 h. The dark purple precipitate was filtered,

washed with ice-water and air dried on the filter. Yield: 61%; GC–EIMS, *m*/*z* [M^+]: 270.1; ^{1}H NMR (300 MHz, DMSO-d_6, rt) δ 7.63 (d, *J* = 9.16 Hz, Ar**H**, 2H), 7.53 (d, *J* = 8.87 Hz Ar**H**, 2H), 6.79 (d, *J* = 9.25 Hz Ar**H**, 2H), 6.64 (d, *J* = 8.88 Hz, Ar**H**, 2H), 2.99 ppm (s, -CH_3, 6H).

Synthesis of *N*,*N*-dimethyl[4-(4-aminophenylazo)phenyl]amine (5)

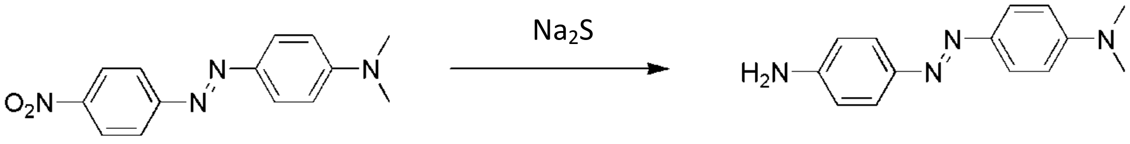

5

Scheme S2: Reduction of the red azo-dye **3**.

To a solution of *N*,*N*-dimethyl-[4-(4-nitro-phenylazo)-phenyl]-amine (2 g, 7 mmol) in 100 mL ethyl acetate, sodium sulfide (3.55 g, 14.8 mmol) was added. The solution was stirred under reflux for 1 h. The solution was cooled down to rt and poured into cold water. The precipitate was filtered, washed with ice-water and air dried on the filter. The dye was purified by column chromatography; eluent: ethyl acetate/ *n*-hexane 1:3. Yield: 80%; GC–EIMS, *m*/*z* [M^+]: 240.1; ^{1}H NMR (300 MHz, DMSO-d_6, rt) δ 7.63 (d, *J* = 9.06 Hz, Ar**H**, 2H), 7.53 (d, *J* = 8.88 Hz, Ar**H**, 2H), 6.79 (d, *J* = 9.26 Hz, Ar**H**, 2H), 6.64 (d, *J* = 8.88 Hz, Ar**H**, 2H), 5.76 (s, NH_2, 2H), 2.99 ppm (s, -CH_3, 6H).

Synthesis and characterization of hyperbranched polyglycerol (HPG)

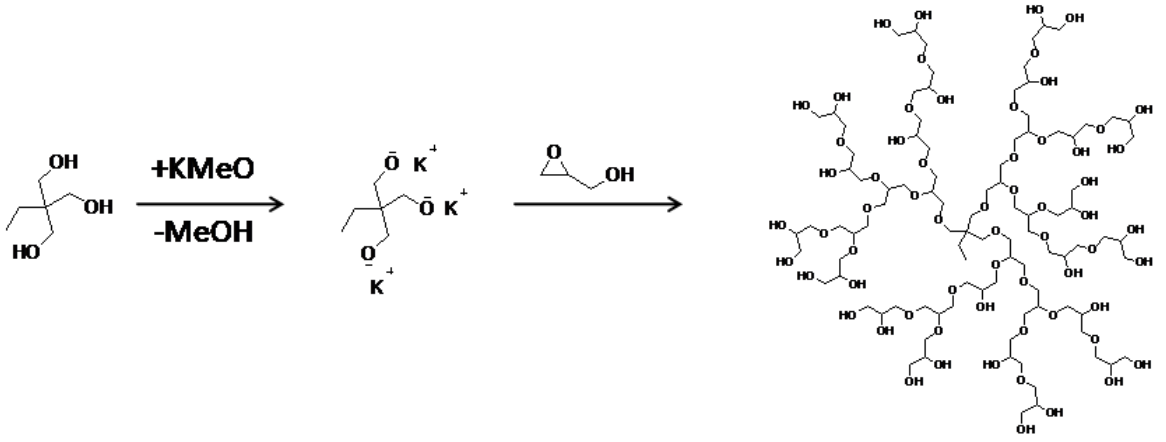

Scheme S3: Synthesis of HPG.

Hyperbranched polyglycerol was synthesized following the preparation method of Frey et al. with an adjusted molar mass of 7 kDa [1]. We achieved a molar mass of M_n = 6.7 kDa with a PD of 1.5 and a DB of 0.57. For determination of the molecular weight, size exclusion chromatography combined with a three-angle static-light-scattering detector was used. The degree of branching was calculated from inversed gated ^{13}C NMR experiments. IR: 3356 (OH), 2870 (CH), 1459 (CH_2), 1068 (COC), 1025 (COH) cm^{-1}; ^{1}H NMR (300 MHz, DMSO-d_6, rt) δ 4.73–4.46 (br, OH), 3.63–3.25 (m, HPG-polymer-backbone), 1.39–1.13 (br, CH3C**H2** initiator), 0.77 ppm (b, C**H3**CH2 initiator); ^{13}C NMR (500 MHz, DMSO-d_6, rt) δ 80.6–79.8 (L13), 78.8–78.0 (D), 73.2–72.6 (L14),72.0–70.6 (D,T), 70–68.7(L13,L14), 63.6–63.1 (T), 61.9–61.0 ppm (L13).

L13 = linear 1,3 linked C-atom; L14 = linear 1,4 linked C-atom; D = dendritic C-atoms; T = terminal C-atom.

Synthesis of propargyl-modified HPG

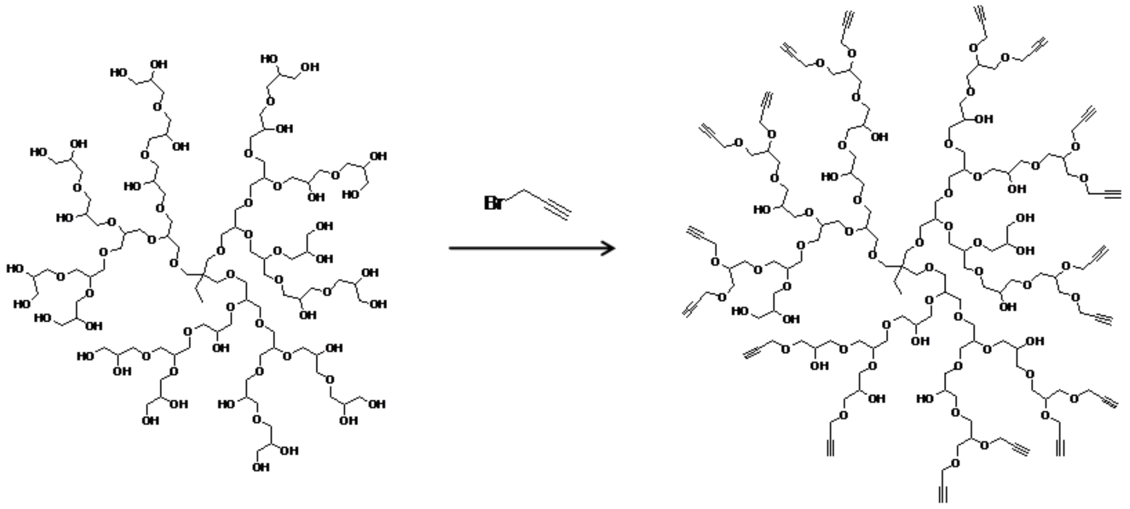

Scheme S4: Synthesis of propargyl-modified HPG.

The propargyl-modified HPG was synthesized according to the synthesis of Haag et al. [2]. IR: 3363 (OH), 3287 (C≡C-H), 2929, 2870 (CH2), 2116 (C≡C), 1458 (CH2), 1348 (OH), 1082 (C-O-C) cm^{-1}; ^{1}H NMR (300 MHz, DMSO-d_6, rt) δ 4.92–4.35 (br, O**H**), 4.35–4.05 (m, -OC$\mathbf{H_2}$CCH), 3.90–3.16 (m, polymer backbone), 1.37–1.20 (b, $CH_3C\mathbf{H}_2$ initiator), 0,80 ppm (b, $C\mathbf{H}_3CH_2$ initiator); ^{13}C NMR (300 MHz, DMSO-d_6, rt) δ 81.3 (-OCH_2C**C**H), 80.15–79.81 (L13), 78.63–77.72 (D), 77.28–76.03 (-OCH_2**C**CH), 73.11–72.80 (L14), 72.04–70.23 (D,T), 70.05–69.03 (L13, L14), 63.33–63.19 (T), 61.40–60.56 (L13), 58.27-57.81 (-O**C**H_2CCH), 57.14-56.76 ppm (-O**C**H_2CCH).

Synthesis of CD-monoazide

Mono(6-azido-6-desoxy)-β-cyclodextrine was synthesized according to the known procedure [3]. IR: 3314 (OH), 2923 (CH), 2102 (N=N), 1365 (OH), 1152 (C-N), 1077 (OH), 1025 (CH) cm^{-1}; ^{1}H NMR (500 MHz, DMSO-d_6, rt) δ 5.72 (m, C-2,-3 OH, 14H),

4.88 (d, 3.3 Hz, C-1, 1H), 4.87 (d, 3.6 Hz, C-1, 6H), 4.42–4.61 (m, C-6 O**H**, 6H), 3.59–3.85 (m, C-2,-3,-5**H**, 28H), 3.33 (m, C-6**H**, 14H); MALDITOF: *m*/*z*: 1182.4 [M + Na^+].

Synthesis of HPG bearing β-CD (7)

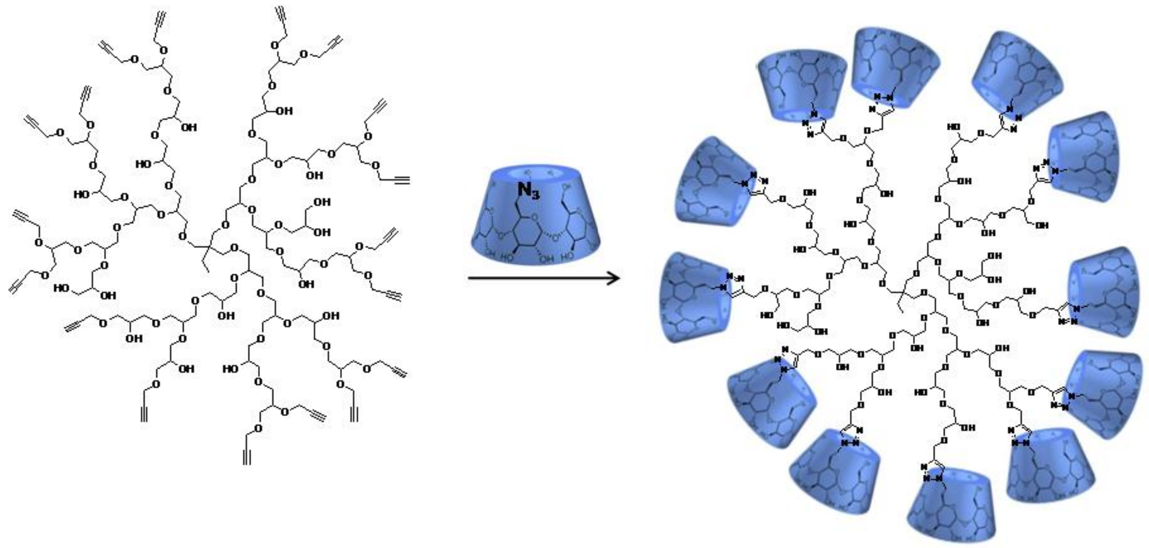

Scheme S5: Synthesis of β-CD modified HPG.

HPG-CD was synthesized by a microwave-assisted “click”-reaction of mono(6-azido-6-desoxy)-β-cyclodextrin with propargyl-modified HPG. 340 mg of propagyl-modified HPG (1.15 mmol in relation to the propargyl moiety), 2 g (2 mmol) CD-monoazide, 29 mg (0.15 mmol) sodium ascorbate and 18 mg (74 µmol) copper(II) sulfate pentahydrate were suspended in 25 mL *N*,*N*-dimethylformamide in a pressure-resistant microwave test tube provided with a magnetic stirring bar. The tube was sealed and placed in the microwave oven and irradiated at 140 °C and 130 W for 60 min. The product was precipitated by addition of acetone, filtered and washed three times with acetone. The crude product was purified by dialysis (regenerated

cellulose, MWCO: 3.5 kDa) in water. The aqueous solution was freeze dried and 1 g of a light-brown solid was obtained. The molar mass of M_n = 38 kDa with a PD = 1.7 was determined by size-exclusion chromatography. The average DS = 30 was proven by ^{1}H NMR spectroscopy, on comparison of the integrated signals at δ 7.95 ppm for the 1,2,3-triazole proton and δ 0.81 ppm for the initiator molecule. IR: 3328 (OH), 2921 (C-H), 1651 (C=C) 1410 (CH), 1332 (OH), 1152 (COH), 1077 (COC), 1027(COH) cm^{-1}; ^{1}H NMR: (500 MHz, DMSO-d_6, rt) δ 8.13 (br C**H**), 6.05–5.45 (br, C-2, C-3O**H**), 5.25–4.20 (br, C1-**H**, C6- O**H,**), 3.90–3.16 (br, C2,C3,C4,C5,C6-**H**, polymerbackbone), 1.36–1.22 (br, $CH_3C\mathbf{H}_2$ Initiator), 0.82 ppm (br, $C\mathbf{H}_3CH_2$ initiator).

Calculation of the molecular weight of 6 by ^{1}H NMR measurements

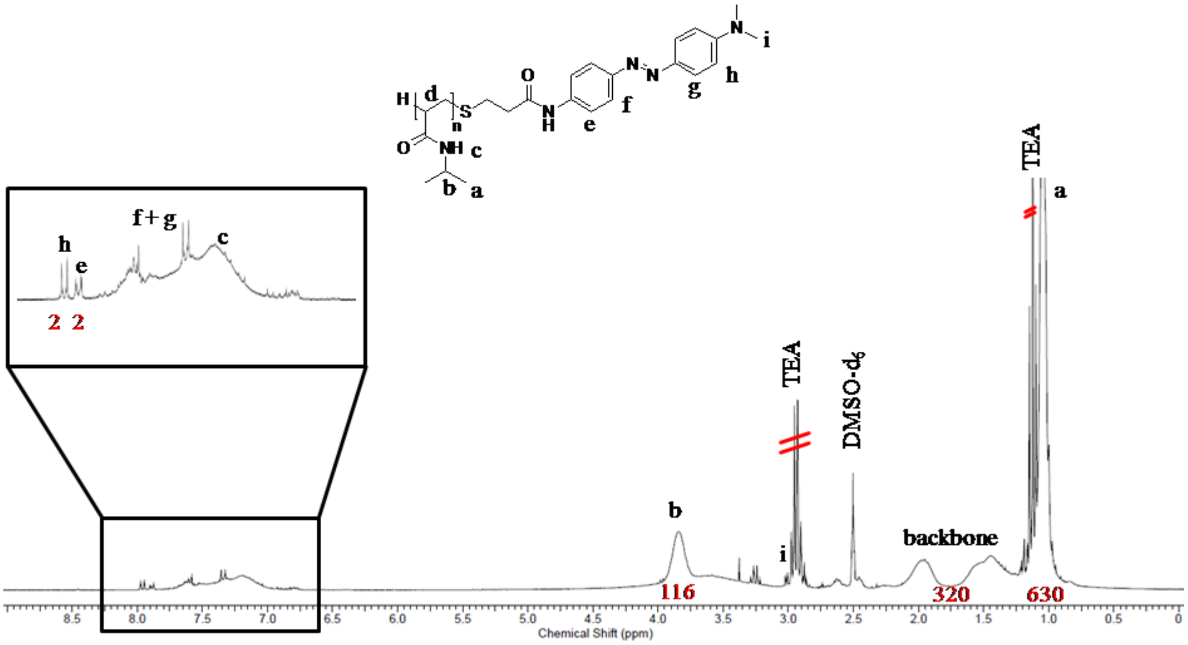

Figure F1: ^{1}H NMR spectrum of **6** in DMSO-d_6.

The molecular weight of **6** was calculated by end-group analysis using the ^{1}H NMR measurements. For the calculations, the ratios of the aromatic azo-dye protons (**h**) (7.9 ppm) and the protons of the single proton of the polymer backbone (**d**) (3.85 ppm) of the PNIPAM-chain were calculated. The value of M_n determined by ^{1}H NMR measurements (M_n = 3.3 kDa) was nearly the same as that determined by the SEC.

2D ROESY NMR spectrum of the complex between 6 and RAMEB-CD

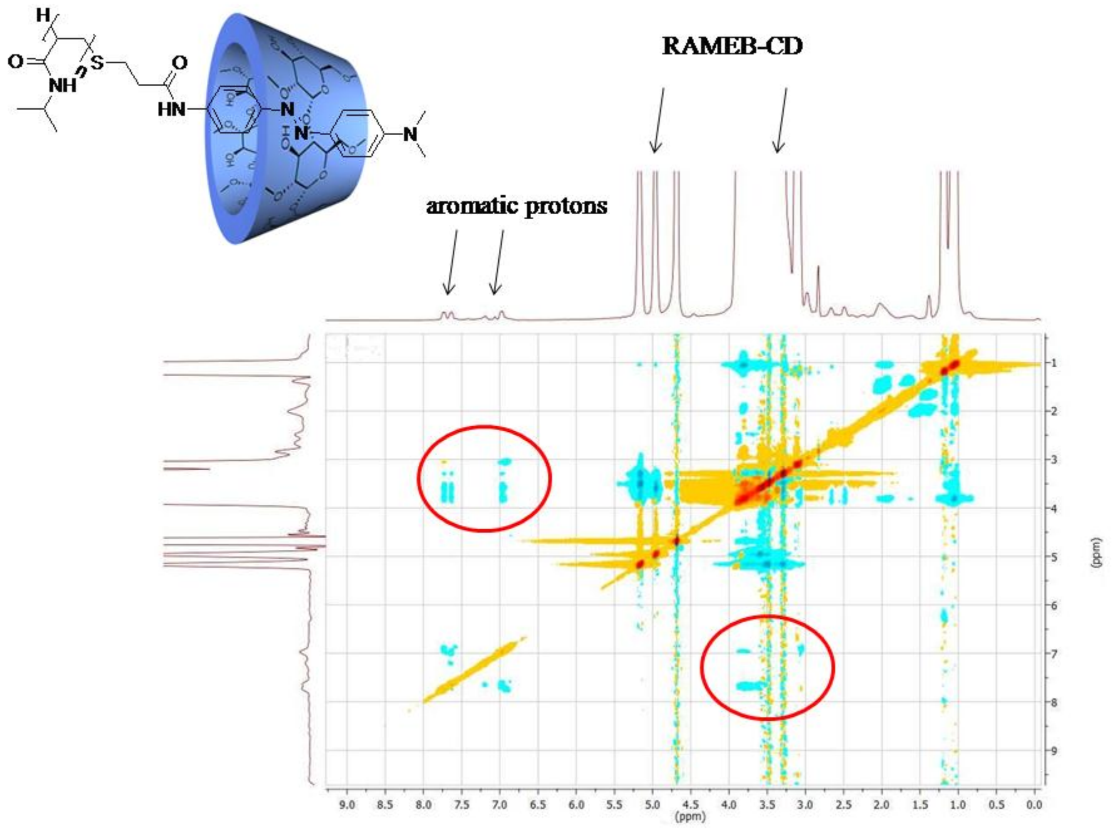

Figure F2: 2D ROESY NMR spectrum of the complex between **6** and RAMEB-CD.

References

1. Sunder, A.; Hanselmann, R.; Frey, H.; Mülhaupt, R. *Macromol.* **1999,** *32,* 4240–4246.

2. Sunder, A.; Mülhaupt, R.; Haag, R.; Frey, H. *Macromol.* **2000,** *33,* 253–254.

3. Liu, H.; Zhang, Y.; Hu, J.; Li, C.; Liu, S. *Macromol. Chem. Phys.* **2009,** *210,* 2125–2137.
